# Improving Pediatric Bronchiolitis Care: Optimizing Nasal Suctioning in Community Emergency Departments

**DOI:** 10.7759/cureus.106285

**Published:** 2026-04-01

**Authors:** Christine E Maloney, Kendra L Powell, Ashley E Keilman, Neil G Uspal

**Affiliations:** 1 Pediatric Emergency Medicine, Seattle Children's Hospital, Seattle, USA; 2 Clinical and Translational Research, Seattle Children's Hospital, Seattle, USA

**Keywords:** community emergency department, continuing health education, pediatric emergency care, simulation-based learning, viral bronchiolitis

## Abstract

Community emergency departments see the majority of children receiving emergency care. However, each of these emergency departments may only see a small volume of pediatric patients per day, often leaving staff uncomfortable when caring for sick infants. By forming partnerships between an academic pediatric emergency department and community emergency departments, we sought to improve the clinical skills of community staff caring for infants with bronchiolitis, a common pediatric presentation. We created a training module that summarizes evidence-based best practices for bronchiolitis management and conducted hands-on suctioning simulations with 31 community emergency department nurses and respiratory therapists. Through education and simulation on bronchiolitis care, the majority of study participants were confident in their ability to improve their care of children with bronchiolitis and intended to change their practice. This multi-center pilot study highlights the power of academic and community emergency department partnerships in enhancing continuing education and promoting pediatric care quality in community emergency departments.

## Introduction

The majority (80-90%) of children receiving emergent care are seen in community emergency departments that care for both pediatric and adult patients [1]. Although the number of children each community emergency department sees varies, the majority see fewer than fifteen pediatric patients per day [2]. Infrequent exposure to pediatric patients contributes to decreased provider comfort and confidence when caring for sick children and performing pediatric procedures [1].

Bronchiolitis is a common respiratory illness primarily affecting children under two years of age and is a leading cause of hospitalization in infants and young children [3]. Supportive care, which includes nasal suction and hydration, is the mainstay of treatment. Most patients with bronchiolitis and moderate-to-severe respiratory symptoms require intermittent nasal suctioning. Nasal suctioning has been shown to improve respiratory scores and oxygen saturation in infants with bronchiolitis [4,5]. Lapses in frequency of suctioning have also been shown to increase inpatient lengths of stay in children with bronchiolitis [6]. Saline drops with mechanical aspiration of the nares help relieve upper airway obstruction, thereby reducing the infants' work of breathing and allowing them to feed and maintain hydration. Despite the benefits of suctioning, the frequency and efficacy of suctioning in the community emergency department setting have not been studied. Education and training regarding care for infants with respiratory distress and suctioning techniques are crucial to improving the comfort of staff caring for this patient population.

Our tertiary children’s hospital has been partnering with regional community emergency departments to support their pediatric readiness. Initial site visits highlighted the desire from community emergency department nurses and respiratory therapists (RTs) to receive additional dedicated training in pediatric bronchiolitis care and suctioning techniques. The primary outcome of this multi-site pilot study is to assess the efficacy of dedicated bronchiolitis education and suctioning simulation to create confidence in community health care providers to change their practice when caring for pediatric patients with bronchiolitis, within a broader partnership between academic pediatric and community emergency departments.

## Materials and methods

Intervention design

We utilized a mixed educational model to review best practices for bronchiolitis care. First, we created a didactic module on bronchiolitis care, highlighting nursing and respiratory therapy interventions for patients with bronchiolitis and respiratory distress. The module was developed using publicly available educational materials on bronchiolitis from ACEP SimBox [7], Colorado Emergency Medical Services for Children [8], and Seattle Children’s Hospital clinical pathways [9]. A summarized example of the module is included in Appendix A. The specific learning objectives addressed in the module were (1) identifying key emergency department nursing and respiratory therapy interventions for patients with bronchiolitis, including suctioning, oxygen delivery as needed, assessment of hydration status, and family and caregiver teaching; (2) understanding suction methods and indications for patients with bronchiolitis; (3) understanding caregiver teaching for patients with bronchiolitis; (4) and demonstrating nasal and nasopharyngeal suction techniques in bronchiolitis patients in the ED.

The module also included education on frequent tests and interventions that are not recommended in the routine care of patients with bronchiolitis, including viral testing, chest X-rays, and treatment with corticosteroids [9].

**Figure 1:**
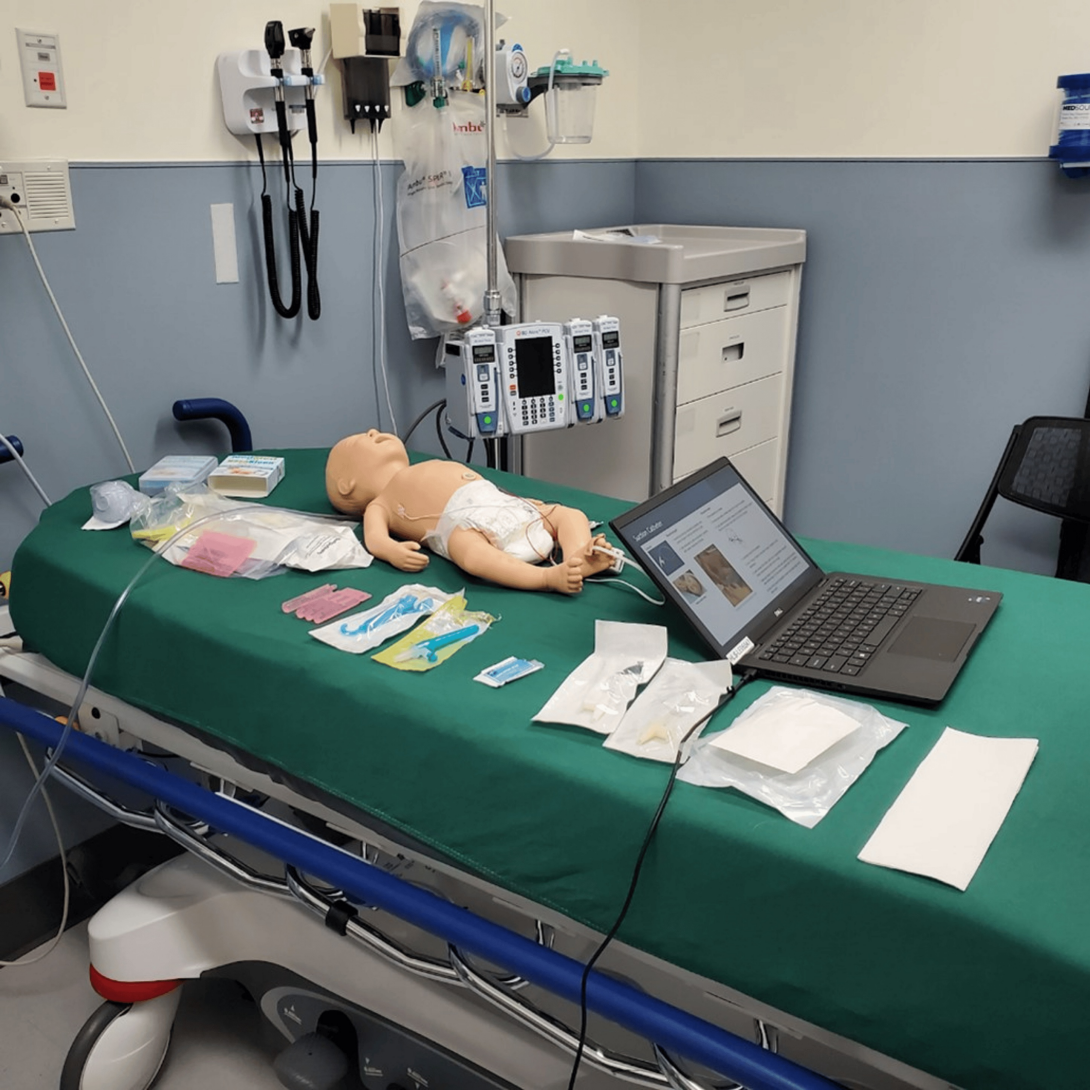
Equipment set-up for simulation practice for suctioning infants with bronchiolitis Image courtesy of the authors.

To accompany the training module, a hands-on simulation session was developed to explore the equipment for suctioning and practice the recommended techniques. This included utilizing manikins for healthcare professionals to practice with different suctioning devices, including olive tip suctioning and nasopharyngeal suctioning. An example of the equipment set-up is shown in Figure 1. A full equipment and supplies list is available in Appendix B.

Study procedures

A nurse educator from Seattle Children's Hospital facilitated the session. Four community emergency departments across Washington state participated in the training session; three are considered general EDs; and one is a satellite ED. These sites see annual pediatric volumes between 1,800 and 10,000 per year. Training sessions began with a statement about the project, the voluntary nature of participation, and an information sheet informing participants about the study. The session began with a didactic portion where the nurse educator reviewed the module content. Participants then had the opportunity to do hands-on training in emergency department patient care areas and conference rooms using low-fidelity manikins. Participants received real-time feedback during hands-on training from the nurse educator. Session duration was approximately one hour. Participants completed surveys before and after the education intervention. The study was conducted between February 12, 2024, and January 5, 2025.

Study participants

Registered nurses (RN), licensed practical nurses (LPN), and RT involved in the emergency department care of pediatric patients were invited to the education sessions. Participation was voluntary, and there was no financial compensation offered.

Data collection

We utilized pre- and post-intervention surveys (Appendix C). The pre- and post-intervention surveys were developed internally by the authors and reviewed for clarity. The pre-survey assessed individuals’ baseline confidence in providing interventions for patients with bronchiolitis. The post-survey evaluated participants’ perceptions of the effectiveness of the curriculum as well as participants' intention to and confidence in altering their practice in caring for children with bronchiolitis. Both surveys asked participants to identify their role in the emergency department and the number of years of experience they had in their roles.

Ethical considerations

The Seattle Children’s Hospital Institutional Review Board (IRB) approved this study prior to its conduct.

## Results

Thirty-one participants from four community emergency departments received the educational intervention (Table 1). Participants represented a broad spectrum of professional experience, spanning early career to highly experienced professionals.

**Table 1:** Participant demographics (n = 31) RN: registered nurse; RT: respiratory therapist.

Participants by site	n (%)
Site 1	3 (10%)
Site 2	10 (32%)
Site 3	5 (16%)
Site 4	13 (42%)
Clinical role	n (%)
RN	29 (94%)
RT	2 (6%)
Years of experience	n (%)
<1 year	8 (26%)
2-5 years	7 (23%)
6-10 years	6 (19%)
>10 years	10 (32%)

Responses to the pre-intervention survey statements are summarized in Table 2. These statements assessed the healthcare workers' confidence in being able to perform each of the following activities: (1) identify key emergency department nursing interventions for patients with bronchiolitis; (2) describe suction methods and indications for patients with bronchiolitis; (3) describe caregiver teaching for patients with bronchiolitis; (4) and demonstrate nasal and nasopharyngeal suction techniques.

**Table 2:** Distribution of responses to Likert-scale survey pre-intervention

Statement: You can confidently perform each activity	Strongly disagree n (%)	Disagree n (%)	Neither agree nor disagree n (%)	Agree n (%)	Strongly agree n (%)	Total n
Identify key emergency department nursing interventions for patients with bronchiolitis	2 (6%)	3 (10%)	13 (42%)	12 (39%)	1 (3%)	31
Describe suction methods and indications for patients with bronchiolitis	2 (6%)	7 (23%)	12 (39%)	8 (26%)	2 (6%)	31
Describe caregiver teaching for patients with bronchiolitis	1 (3%)	11 (36%)	11 (36%)	6 (19%)	2 (6%)	31
Demonstrate nasal and nasopharyngeal suction techniques	2 (6%)	12 (39%)	8 (26%)	8 (26%)	1 (3%)	31

Table 3 summarizes responses categorized by years of experience.

**Table 3:** Mean Likert-scale pre-intervention survey by years of experience

	Mean Likert response level (1-5) pre-intervention by years of experience			
Statement: You can confidently perform each activity	<1 year (n = 8)	2-5 years (n = 7)	5-10 years (n = 6)	>10 years (n = 10)
Identify key emergency department nursing interventions for patients with bronchiolitis	2.5	3.4	3.2	3.7
Describe suction methods and indications for patients with bronchiolitis	2.4	2.7	3.7	3.4
Describe caregiver teaching for patients with bronchiolitis	2.4	2.4	3.7	3.2
Demonstrate nasal and nasopharyngeal suction techniques	2.3	2.3	3.5	3.2

Table 4 summarizes participant responses from the post-intervention survey. In this survey, participants were asked if the activity supported the achievement of each learning objective, if they intended to change practice behavior, and how confident they felt they could make the intended changes. The majority of participants agreed or strongly agreed that the intervention supported the intended learning objectives.

**Table 4:** Distribution of responses to Likert-scale survey post-intervention

Statement: The activity supported the achievement of the learning objective	Strongly disagree n (%)	Disagree n (%)	Neither agree nor disagree n (%)	Agree n (%)	Strongly agree n (%)	Total n
Identify key emergency department nursing interventions for patients with bronchiolitis	0 (0%)	0 (0%)	2 (6%)	8 (26%)	21 (68%)	31
Describe suction methods and indications for patients with bronchiolitis	0	0	2 (6%)	4 (13%)	25 (81%)	31
Describe caregiver teaching for patients with bronchiolitis	0 (0%)	1 (3%)	1 (3%)	6 (19%)	23 (74%)	31
Demonstrate nasal and nasopharyngeal suction techniques	0 (0%)	1 (3%)	1 (3%)	9 (29%)	20 (65%)	31

Table 5 summarizes mean responses categorized by years of experience.

**Table 5:** Mean Likert-scale responses post-intervention by years of experience

	Mean Likert response level (1-5) post-intervention by years of experience			
Statement: The activity supported the achievement of the learning objective	<1 (n = 8)	2-5 years (n = 7)	5-10 years (n = 6)	>10 years (n = 10)
Identify key emergency department nursing interventions for patients with bronchiolitis	4.2	4.9	5	4.6
Describe suction methods and indications for patients with bronchiolitis	4.4	4.9	5	4.8
Describe caregiver teaching for patients with bronchiolitis	4	4.9	5	4.8
Demonstrate nasal and nasopharyngeal suction techniques	3.9	4.7	5	4.7

A total of 90% of participants stated that they intended to change their practice following the training, with 93% stating they agreed or strongly agreed that they had confidence they would be able to make the intended changes. In free-text responses, participants indicated changes they intended to make to include suctioning first, more education to families (including guidance on at-home suctioning practices and devices and encouraging this before feeding and sleeping), avoiding nebulized treatments, and chest X-rays. Nursing colleagues expressed a plan to provide nasal suctioning prior to respiratory therapy arrival or when respiratory therapy is unavailable.

## Discussion

This multi-site pilot study of an educational intervention in community emergency departments demonstrates effectiveness in creating intention to and confidence in changing clinical practice in nurses and RTs managing pediatric patients with bronchiolitis.

There are many interventions in bronchiolitis that are considered low-value, including bronchodilators, steroids, and hypertonic saline [10]. However, these low-value interventions continue to be utilized in large part because, despite the lack of evidence in their efficacy, emergency department staff perceive them as beneficial. When assessing factors that lead staff to trial a bronchodilator, cited factors included presence of wheezing on exam, personal or family history of wheezing, oxygen saturation, parental concern/anxiety, and “concern the healthcare personnel are doing nothing” [11]. Notably, nurses and RTs have perceived greater benefit than physicians/licensed practitioners from these therapies. This highlights the importance of designing educational interventions with an interprofessional mindset and supports inclusion of a review of these low-value interventions during continuing education efforts.

The mainstay of treatment for bronchiolitis is supportive care, which can be difficult to explain to caregivers. Prior qualitative studies on caregiver perspectives in managing their child with bronchiolitis included themes such as feeling unprepared, being afraid to discharge home after seeing their child’s health deteriorate quickly, and lacking information and understanding, resulting in a sense of helplessness [12]. Training families in the use of appropriate suctioning devices has been associated with decreased resource utilization and improved family satisfaction post emergency department discharge [13]. Empowering caregivers to understand their child’s illness and framing home suctioning as an intervention, or “prescription,” they can carry out at home gives them an actionable way to support their child’s care. Additionally, reviewing the expected clinical course of viral illness, highlighting that symptoms may worsen if their child is early on in their illness, gives caregivers a sense of what to expect and return precautions for when to seek repeat evaluation. Nurses and RTs who participated in this learning module felt the module supported their understanding of caregiver teaching for patients with bronchiolitis.

When reviewing suctioning techniques, a multi-disciplinary session was of high value as RTs and nurses provided unique perspectives to each other on tips and tricks for suctioning pediatric patients. It promoted meaningful interdisciplinary conversations about the scope of care and roles and responsibilities. This intervention also facilitated a review of available suctioning equipment, including hospital-specific inventory, storage locations, and the need for potential equipment modifications.

Participants in this intervention brought varied professional backgrounds and experience, ranging from less than one year of experience to over 10 years of experience. Despite varying experience levels, all groups expressed an intent to change practice behavior when caring for patients with bronchiolitis, highlighting the importance of offering this training to all staff. Continuing education efforts are essential, as even seasoned healthcare personnel can benefit from updated knowledge and practicing skills that may be used infrequently [1].

This study highlights the broader benefits of partnership between academic children’s hospitals and community emergency departments in enhancing continuing education and support when caring for ill children. Disseminating current evidence and procedural best practices to community sites, where the majority of pediatric patients receive care, is crucial to ensuring children have access to quality emergency care, regardless of where they present [14]. Studies have shown variability in pediatric emergency care across care settings, likely due to delayed adoption of pediatric best practices, familiarity with pediatric bedside procedures, and other factors [15]. For example, the use of chest X-ray in respiratory diseases, including asthma, bronchiolitis, and croup, has been shown to be lower in pediatric-specific EDs when compared to general EDs [16]. A focus on the dissemination of pediatric best practices and how these are adapted at the community emergency department level is crucial.

Study limitations

As this is a pilot study with voluntary participation, the sample size is small. Participants also self-selected for participation, potentially skewing survey results. Our study only measured intention to change practice and not actual change in practice. We do not know if the intervention resulted in an actual change in practice. Additional limitations include the absence of a control group or alternative educational intervention to compare our intervention to, as well as the potential for response bias in evaluations from the study participants. To minimize the risk of response bias, evaluations were anonymous and not reviewed until after the event. Suctioning equipment and protocols varied at each site and were not changed by the study team. Local best practices were reviewed at all sites, and participants practiced utilizing available hospital-specific equipment during simulation.

While the nurse educator from the academic children’s hospital did receive funding for their time during this initial pilot study, this intervention was designed to be implementable by educators at community emergency departments and adaptable for additional sites at low cost. The training module was developed from pre-existing, publicly available educational materials. The educational module used in this intervention is included in the appendices to facilitate adoption of this intervention in community emergency departments. The manikin was low fidelity, and training supplies were pulled from expired clinical equipment. While employee time varied at each site, for many, this was built into existing continuing education training.

Future considerations

Future considerations would be to assess the effectiveness and feasibility of a train-the-trainer model to continue educational sessions as staff turnover occurs at sites. Additional evaluation to assess the effectiveness of this educational intervention on impacting clinical outcomes for children with bronchiolitis and potentially reducing unnecessary transfers to higher levels of care is warranted. This training could be expanded to include providers in addition to nurses and RTs to encourage a cohesive and multidisciplinary approach to care.

## Conclusions

Through a partnership between an academic children’s hospital and community emergency departments, we designed and implemented an educational intervention on bronchiolitis care for nurses and RTs. The majority of participants reported an intention to change their clinical practice as a result of the intervention. These results highlight the value of these partnerships to disseminate best practices in pediatric care and improve confidence and readiness among community emergency department teams caring for pediatric patients.

## Figures and Tables

**Figure 2:**
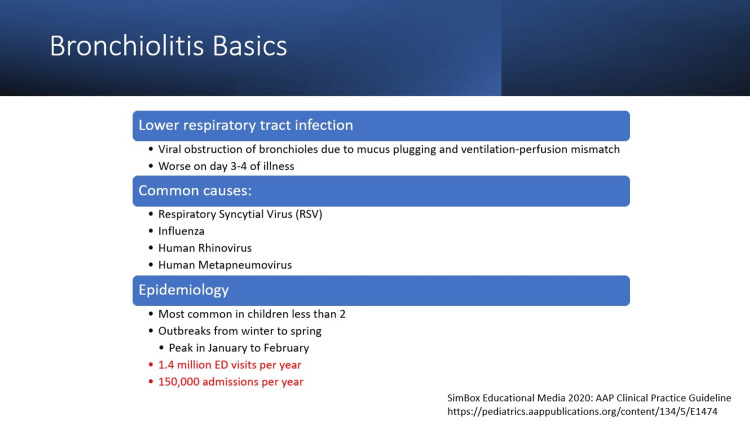
Slide 2 Image created independently by authors based on content from [7].

**Figure 3:**
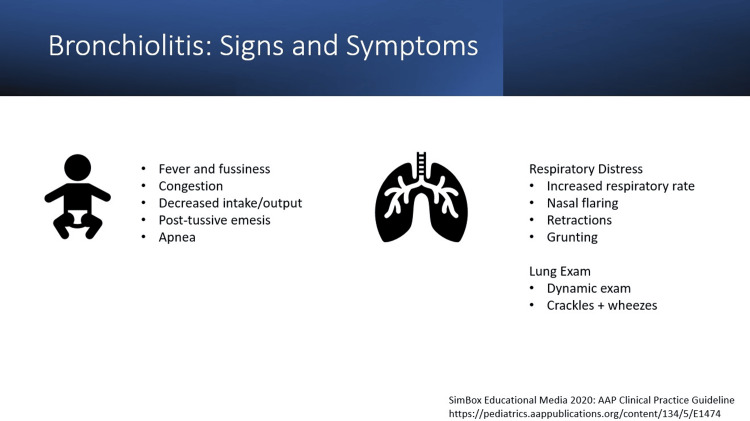
Slide 3 Image created independently by authors based on content from [7].

**Figure 4:**
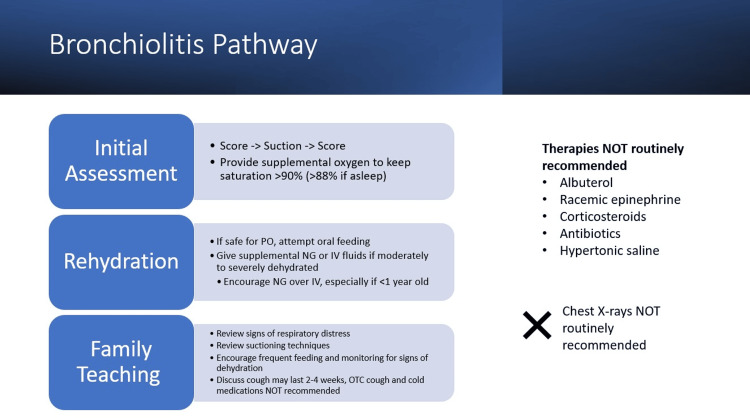
Slide 4 Image created independently by authors based on content from [9].

**Figure 5:**
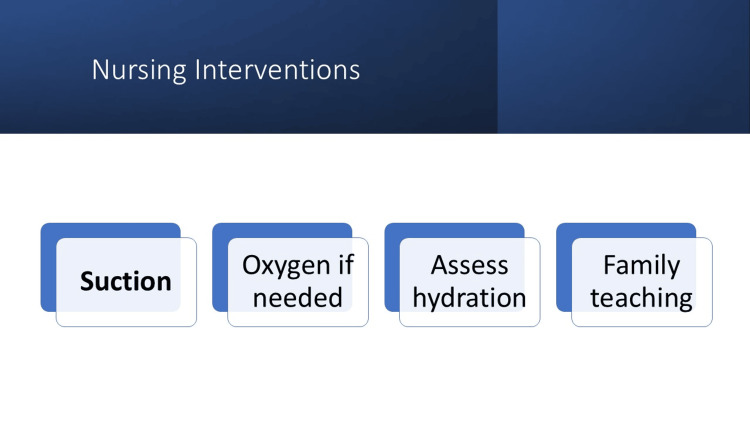
Slide 5

**Figure 6:**
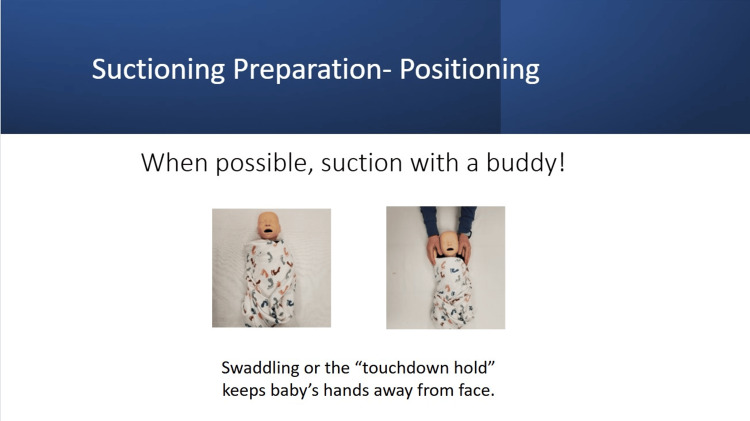
Slide 6

**Figure 7:**
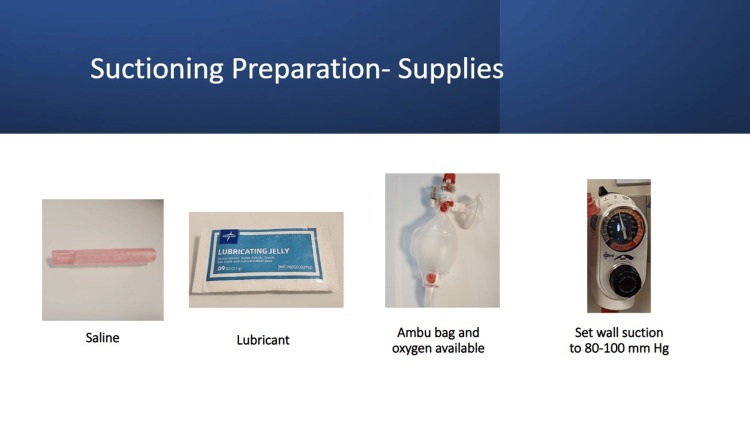
Slide 7

**Figure 8:**
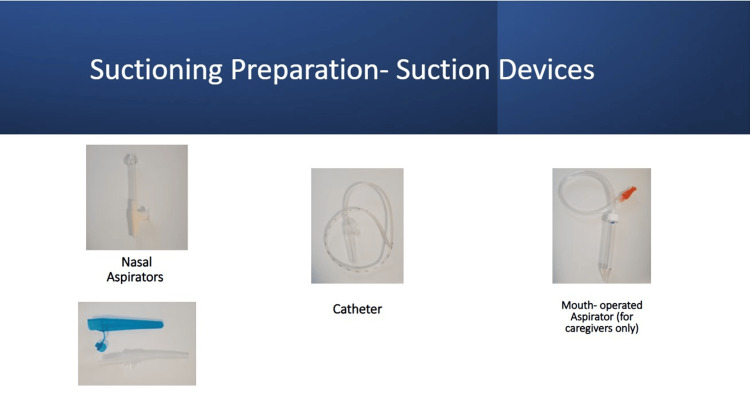
Slide 8

**Figure 9:**
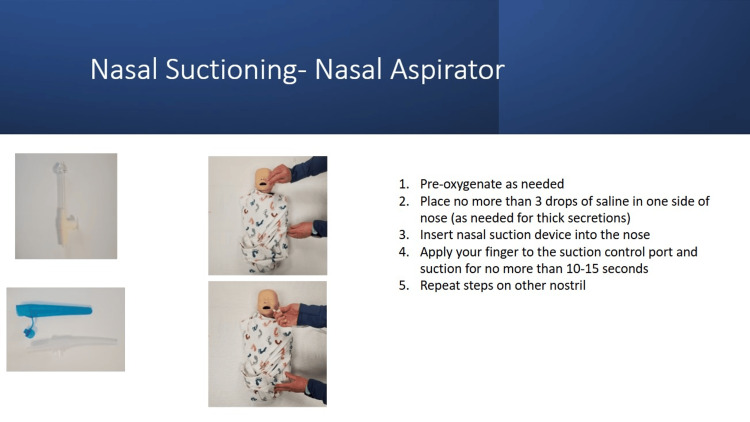
Slide 9

**Figure 10:**
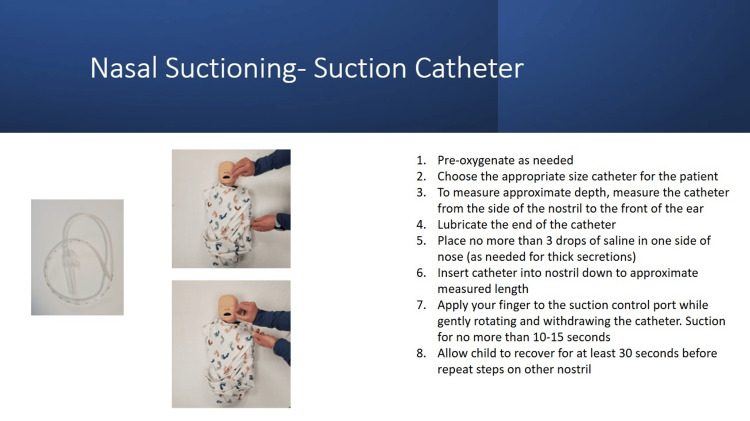
Slide 10

**Figure 11:**
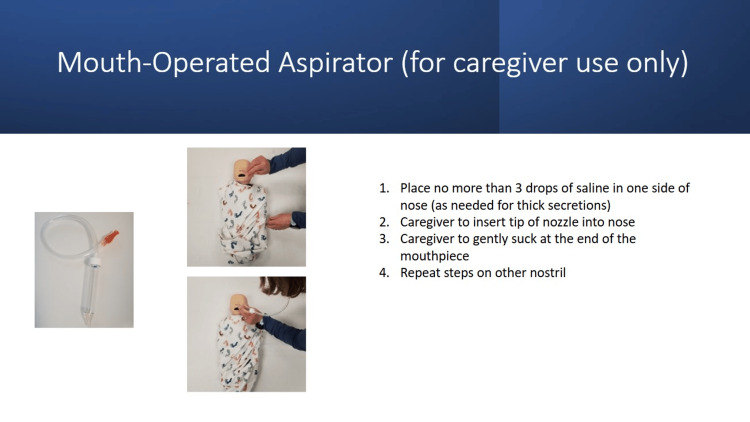
Slide 11 Images created independently by authors based on content from [17].

**Figure 12:**
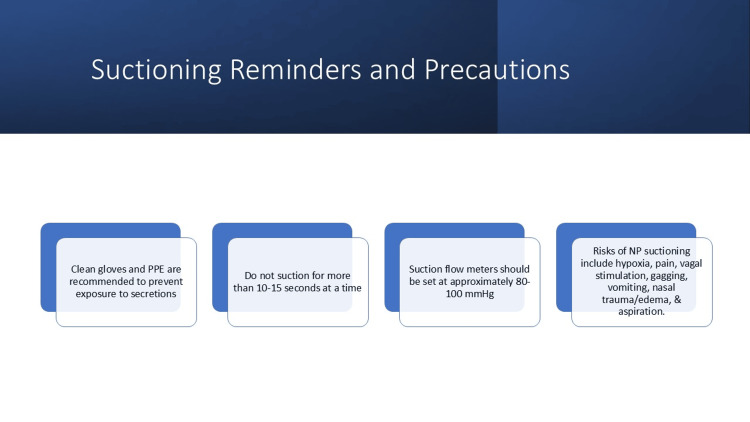
Slide 12

## Appendices

Appendix A

Below (Figures 2-13) is a summary of the didactic materials utilized for the educational component.

Appendix B

A full equipment list for the educational sessions is provided in Table 6.

**Table 6:** Pediatric suctioning simulation equipment list

Pediatric suctioning simulation equipment list
AV equipment for sharing the PowerPoint module (laptop at some locations; computer and screen at others, where available)
Infant manikin with patent nares
Bulb syringe
Nasal suction devices (olive tip, mushroom tip, boogie baby, and little sucker)
Suction catheters (8Fr)
Saline fish
Lubricant
Nasal aspirators for home use to demonstrate caregiver suctioning at home
Wall suction regulator (provided by site in situ)
Suction tubing (provided by site in situ)
Printed surveys for staff (pre- on one side; post- on the other)
Pencils for staff surveys

Appendix C

Below includes the full pre-intervention (Table 7) and post-intervention (Table 8) surveys participants were asked to fill out. 

**Table 7:** Pre-intervention survey RN: registered nurse; RT: respiratory therapist.

Pre-survey
What is your current practice when a patient with bronchiolitis presents with respiratory distress?
Please indicate the extent to which you agree/ disagree that you can confidently perform each activity. Circle one for each of the following: (1 strongly disagree-5 strongly agree)
Identify key emergency department nursing interventions for patients with bronchiolitis
1 2 3 4 5
Describe suction methods and indications for patients with bronchiolitis
1 2 3 4 5
Describe caregiver teaching for patients with bronchiolitis
1 2 3 4 5
Demonstrate nasal and nasopharyngeal suction techniques
1 2 3 4 5
What is your role?
RN
MD
RT
APP
Other
How many years of experience do you have in your role?
<1 year
2-5 years
6-10 years
>10 years

**Table 8:** Post-intervention survey RN: registered nurse; RT: respiratory therapist.

Post-survey
Please indicate the extent to which you agree/disagree that the activity supported the achievement of each learning objective. Circle one for each of the following: (1 strongly disagree-5 strongly agree)
Identify key emergency department nursing interventions for patients with bronchiolitis
1 2 3 4 5
Understand suction methods and indications
1 2 3 4 5
Understand caregiver teaching for patients with bronchiolitis
1 2 3 4 5
Demonstrate nasal and nasopharyngeal suction techniques
1 2 3 4 5
Do you intend to change your practice behavior when a patient with bronchiolitis presents with respiratory distress?
Yes/No/Unsure
If you plan to change your practice behavior, what types of changes do you plan to implement?
Please indicate the extent to which you agree/disagree that you are confident that you will be able to make your intended changes. Circle (1 strongly disagree-5 strongly agree):
1 2 3 4 5
What is your role?
RN
MD
RT
APP
Other
How many years of experience do you have in your role?
<1 year
2-5 years
6-10 years
>10 years
